# Exploring the Genomic Landscape of *Bacillus paranthracis* PUMB_17 as a Proficient Phosphatidylcholine-Specific Phospholipase C Producer

**DOI:** 10.3390/cimb46030158

**Published:** 2024-03-14

**Authors:** Vesselin Baev, Ivan Iliev, Yordan Stefanov, Marinela Tsankova, Mariana Marhova, Elena Apostolova, Mariyana Gozmanova, Galina Yahubyan, Sonya Kostadinova

**Affiliations:** 1 Department of Molecular Biology, Faculty of Biology, University of Plovdiv, Tzar Assen 24, 4000 Plovdiv, Bulgaria; eapostolova@uni-plovdiv.bg (E.A.); mariank@uni-plovdiv.bg (M.G.); gyahubyan@uni-plovdiv.bg (G.Y.); 2 Department of Biochemistry and Microbiology, Faculty of Biology, University of Plovdiv, Tzar Assen 24, 4000 Plovdiv, Bulgaria; iziliev@uni-plovdiv.bg (I.I.); marinela_89@uni-plovdiv.bg (M.T.); marhova@uni-plovdiv.bg (M.M.); skosta@uni-plovdiv.bg (S.K.); 3 Biovet JSCo, Petar Rakov 39, 4550 Peshtera, Bulgaria; iordanstefanov@gmail.com

**Keywords:** *Bacillus paranthracis*, genomics, genome annotation, nanopore sequencing, genome sequencing, genome annotation, PC-PLC, enzyme activity

## Abstract

Phospholipases find versatile applications across industries, including detergent production, food modification, pharmaceuticals (especially in drug delivery systems), and cell signaling research. In this study, we present a strain of *Bacillus paranthracis* for the first time, demonstrating significant potential in the production of phosphatidylcholine-specific phospholipase C (PC-PLC). The investigation thoroughly examines the *B. paranthracis* PUMB_17 strain, focusing on the activity of PC-PLC and its purification process. Notably, the PUMB_17 strain displays extracellular PC-PLC production with high specific activity during the late exponential growth phase. To unravel the genetic makeup of PUMB_17, we employed nanopore-based whole-genome sequencing and subsequently conducted a detailed genome annotation. The genome comprises a solitary circular chromosome spanning 5,250,970 bp, featuring a guanine–cytosine ratio of 35.49. Additionally, two plasmids of sizes 64,250 bp and 5845 bp were identified. The annotation analysis reveals the presence of 5328 genes, encompassing 5186 protein-coding sequences, and 142 RNA genes, including 39 rRNAs, 103 tRNAs, and 5 ncRNAs. The aim of this study was to make a comprehensive genomic exploration that promises to enhance our understanding of the previously understudied and recently documented capabilities of *Bacillus paranthracis* and to shed light on a potential use of the strain in the industrial production of PC-PLC.

## 1. Introduction

The identification of phospholipase C (PLC) holds significant importance in bacterial pathogenesis, as the development of inhibitors for these enzymes may yield potential vaccines and therapeutic agents, thereby mitigating the impact of associated diseases in animals and humans [1]. PLC exhibits diverse physiological functions, participating in nutrient substrate provisioning, membrane maintenance and remodeling, and the regulation of cellular mechanisms by generating bioactive lipid molecules. The hydrolysis of phospholipids generates bioactive molecules, such as diacylglycerol, phosphatidic acid, lysophosphatidic acid, and arachidonic acid, participating in various physiological and pathophysiological processes such as membrane transport, cell proliferation, signal transduction, and apoptotic cell damage [1,2,3].

The hydrolysis of phospholipids plays a pivotal role in the bacterial invasion of host cells and cell lysis. Some bacterial PLCs act as pathogenicity factors, constituting active components of bacterial toxins, while also contributing to phagosome evasion, tissue colonization, infection progression, and immune response suppression [1,4,5]. In eukaryotic cells, disrupted phospholipase regulation may contribute to a range of physiological deviations, designating these enzymes as therapeutic targets for both prevention and treatment [6].

The interaction of PLCs with membrane phospholipids can be leveraged to study the phospholipid composition of membranes or faithfully replicate the action of eukaryotic PLCs on cellular metabolism [7]. Microbial PLCs, particularly those derived from *Bacillus* species, have garnered more extensive research attention due to their ease of cultivation and greater availability compared to those from plant seeds and mammals. Due to their distinct structure and substrate preferences and requirements, these enzymes have emerged as versatile biocatalysts with considerable significance. The *Bacillus* species, in particular, have been the focal point of PLC research, given the simplicity of detecting their secreted PLCs and their catalytic activity through relatively straightforward procedures.

The *Bacillus cereus* group encompasses at least 21 interrelated species, spanning a spectrum from pathogens to probiotics, as documented in seminal works [8,9,10]. Among these species, *Bacillus paranthracis*, characterized as Gram-stain-positive, facultatively anaerobic, non-motile, and rod-shaped bacteria, represents a notable entity isolated from diverse environments, including the rhizosphere and soil [11]. Despite its ecological prevalence, knowledge about this organism is scarce, as its formal classification was only introduced in 2017 by Liu and coworkers [10]. This taxonomic delineation was founded on the assessment of Average Nucleotide Identity (ANI) values, coupled with the consideration of physiological and biochemical attributes exhibited by the respective strains. So far, there is a small number of papers that report a comprehensive analysis of the genomic profile of *Bacillus paranthracis*.

Here, we describe a *Bacillus paraanthracis* strain, designated as PUMB_17, for the first time, showing significant promise in the production of phosphatidylcholine-specific phospholipase C (PC-PLC). This study covers a comprehensive analysis of the activity of PC-PLC and its purification. Furthermore, to elucidate the genomic landscape of PUMB_17, we used nanopore-based whole-genome sequencing and subsequently annotated the genome. The goal is to make a systematic genomic study that will facilitate a deeper understanding of the hitherto understudied and recently documented attributes of *Bacillus paranthracis* and, further, to make a preliminary investigation of the strain’s PC-PLC production abilities.

## 2. Materials and Methods

### 2.1. Bacterial Strain, Culturing, and PC-PLC Screening Conditions

The strain PUBM_17, isolated from soil, belongs to the laboratory collection housed within the “Biochemistry and Microbiology” Department at the University of Plovdiv “Paisii Hilendarski”, Bulgaria. Initial taxonomic classification as a *Bacillus* sp. was established through scrutiny of morphological and cultural characteristics. The strain has been identified as Gram-positive, forming thermoresistant spores that are centrally located and do not distend sporangia. It exhibits an aerobic nature with a respiratory metabolism and is classified as a chemoorganotroph.

PC-PLC activity was initially discerned through cultivation on lecithin agar [12]. Petri dishes were incubated for 24 h at 37 °C and the qualitative assessment of PC-PLC production was based on the formation of diglyceride zones surrounding bacterial colonies. The quantitative analyses of PC-PLC production in *Bacillus* sp. PUBM_17 was conducted following deep cultivation of the strain in Gerasimene medium [13] at 37 °C on a rotary shaker (100 rpm). To delineate the dynamics of PC-PLC production, samples were taken from the cultural medium at 2 h intervals. These samples were analyzed for enzymatic activity, pH, and cell density (McFarland Standard, McF).

### 2.2. DNA Extraction, Sequencing Library Generation, Sequencing, and Assembly

DNA was extracted from the PUBM_17 isolate using the QIAamp DNA Microbiome Kit (QIAGEN, Hilden, Germany). The quantity and integrity of the DNA were evaluated using a Qubit 4 fluorometer (Thermo Fisher Scientific, Waltham, MA, USA) and agarose gel electrophoresis, respectively.

For the long-read ONT library preparation, Ligation Sequencing Kit SQK-RBK114 (Oxford Nanopore Technologies, Oxford, UK) was employed with 1 μg of total DNA following the manufacturer’s protocol. Subsequently, the library was sequenced on a MinION using an R10 flow cell (Oxford Nanopore Technologies, Oxford, UK). Base calling and quality control were conducted offline using Guppy v6.5.7 (Oxford Nanopore Technologies, Oxford, UK). Adapter trimming was carried out using Porechop v.0.2.4 with default parameters (https://github.com/rrwick/Porechop, 12 March 2024).

De novo assembly was executed using Flye v2.9.2 with default parameters, excluding reads shorter than 1000 bp [14]. Assembly polishing was achieved through the Racon v1.4.21 (https://github.com/isovic/racon, 12 March 2024) and Medaka v1.8.1 (https://github.com/nanoporetech/medaka, 12 March 2024) tools. The quality of the assembled sequence was evaluated using the CheckM v1.1.6 tool [15,16]. A circular genome map was generated from the single circular chromosome contig using the Proksee tool (https://proksee.ca/, 12 March 2024). The complete genomic sequences of *B. paranthracis* PUMB_17 have been deposited in the NCBI submission portal under accession numbers CP129604 (chromosome), CP129603, and CP129602 (plasmids).

### 2.3. Genome-Based Strain Identification

For bacterial species identification, the PUBM_17 isolate’s ANI was computed using FastANI v 1.34 [17]. A whole-genome sequence-based phylogenetic tree was generated using the Type (Strain) Genome Server (TYGS) (https://tygs.dsmz.de/, 12 March 2024) [18].

### 2.4. Genome Annotation

The PUMB_17 genome sequence was submitted to NCBI Genomes and accession numbers were assigned. Subsequently, the resultant GenBank file underwent annotation by the Rapid Annotations using Subsystems Technology (RAST) web server [19]. Additionally, functional annotations were conducted using the KEGG database and BlastKOALA tool [20].

The PUMB_17 genome was scanned for antimicrobial resistance (AMR) and virulence (VF) genes using the Abricate tool v1.0.1 (https://github.com/tseemann/abricate, 12 March 2024) with default parameters against the Comprehensive Antibiotic Resistance Database (CARD) [21], MEGARes DB [22,23], and the Virulence Factor of Bacterial Pathogen database (VFDB) [24]. AMR annotation was further enriched with entries from the BlastKOALA tool. Prediction of carbohydrate-active enzymes (CAZymes) was carried out using the dbCAN server [25].

### 2.5. Quantitative Determination of PC-PLC Activity

This method is based on the quantitative determination of inorganic, acid-soluble phosphate in phosphorylcholine, released during enzymatic hydrolysis [26]. The reaction mixture contained 200 µL of L-α-phosphatidylcholine (99% from egg yolk, Merck KGaA, Darmstadt, Germany), 200 µL 0.2 M Na_2_B_4_O_7_-HCl buffer (Merck KGaA, Darmstadt, Germany), pH 7.2, and 100 µL enzyme sample. The enzyme hydrolysis reaction was incubated for 10–20 min at 37 °C and was stopped by adding 100 µL 60% HClO_4_ (Merck KGaA, Darmstadt, Germany). The phosphate was extracted via liquid–liquid extraction using 2.5 mL of extraction solution (chloroform–methanol–HCl at 66:33:1) (Merck KGaA, Darmstadt, Germany). After vortexing for 60 s, the mixture was centrifuged for 10 min at 2000 rpm. In total, 200 µL sample was taken from the upper methanol–water layer and hydrolyzed in 700 µL of 60% HClO_4_ for 80 min at 170 °C. Then, 3 mL of ultrapure H_2_O was added to the hydrolyzed samples, followed by incubation at 100 °C for 10 min. To the cooled samples, 500 µL 2.5% ammonium molybdate, 50 µL 1% Triton X100 (Merck KGaA, Darmstadt, Germany), and 1 mL ultrapure H_2_O were added. The reaction mixture was incubated at room temperature for 35 min and, after that, the absorbance at 510 nm was measured. One PC-PLC unit equals the amount of enzyme needed to release 1 µmol phosphate per minute at pH 7.2 and 37 °C.

### 2.6. PC-PLC Purification

#### 2.6.1. Ultrafiltration

The culture broth of *Bacillus* sp. PUBM_17 was centrifuged for 20 min at 14,000× *g* at 4 °C to obtain a cell-free supernatant. It was initially concentrated via ultrafiltration using an Amicon^®^ Stirred cell (Millipore Merck KGaA, Darmstadt, Germany) system and membrane Ultracel filters with a cutoff of 10 kDa (Millipore Merck KGaA, Darmstadt, Germany), under constant pressure (0.5 atm) using argon gas at room temperature.

#### 2.6.2. Size Exclusion Chromatography

The protein concentrate obtained after ultrafiltration was subjected to size exclusion chromatography (SEC). The separation of proteins according to their molecular mass was performed using a glass column (94 × 2.5 cm) filled with Sephadex G-75 (Cytiva, Marlborough, MA, USA) as the stationary phase and 0.05 M Tris-HCl pH 7.8 (Merck KGaA, Darmstadt, Germany) as the mobile phase. Eluted proteins are collected in fractions with a volume of 7 mL each and a flow of 15 mL/h.

#### 2.6.3. Anion Exchange Chromatography (AEX)

An enzyme preparation of phospholipase C after SEC was centrifuged at 10,000× *g* at 4 °C for 20 min. The supernatant was collected and filtered using a 0.22 µm filter to remove large particles. The sample was applied on a HiPrep DEAE FF16/10 (GE Healthcare, Chicago, IL, USA) prefilled with DEAE Sepharose Fast Flow as the stationary phase. The column was equilibrated with 2 column volumes of 0.025 M Tris-HCl, pH 8 buffer. A gradient (0 → 1 M NaCl, 0.025 M Tris-HCl, pH 8, 4.5 column volumes) with a constant flow of 18 mL/h was applied for the elution of the protein. Fractions with a volume of 3 mL were collected. The AEX purification was carried out on an AKTA FPLC system (GE Healthcare, Chicago, IL, USA) equipped with a UPC-900 UV detector and Frac-920 fraction collector. The collected data were analyzed using Unicorn Control Software 7 (GE Healthcare, Chicago, IL, USA).

### 2.7. Protein Concentration Quantification

Protein concentration was calculated at each step via the Bradford method [27], using bovine serum albumin (BSA, Merck KGaA, Darmstadt, Germany) as the standard. The Lowry protein assay, as modified by the Hartree assay [28], was periodically used as confirmatory analysis for protein concentration calculated using the Bradford procedure.

### 2.8. SDS–PAGE Electrophoresis and PC-PLC Zymography

Polyacrylamide gel electrophoresis (PAGE) with 50 µL properly diluted protein samples was performed in the presence of sodium dodecyl sulphate (SDS) using 10% acrylamide with a stacking gel containing 6% acrylamide, as described by Laemmli [29]. Images were generated with Gel Doc EZ Imager (Bio-Rad Laboratories, Hercules, CA, USA). Image Lab™ software (Bio-Rad Laboratories, USA) was used for the Molecular weight analyses. A low molecular weight calibration Kit—14.4–97 kD (Cytiva, Marlborough, MA, USA) was used for the molecular weight determination of the proteins. For zymography, the proteins were renatured via incubation in a Triton-X100 (Merck KGaA, Darmstadt, Germany) solution for 30 min at room temperature. The gel was directly placed on egg yolk agar plate [30]. The PC-PLC activity of the separated proteins was detected, after overnight incubation at 37 °C, as an opaque band formed as a result of lecithin hydrolysis.

### 2.9. Statistical Analysis

STATISTICA version 12 (StatSoft, Dell, TX, USA) was used for statistical analysis and graphical representation of the results.

## 3. Results and Discussion

### 3.1. Extracellular Production of PC-PC by Bacillus sp. PUMB_17

In the initial screening on lecithin agar, notable PC-PLC activity was observed in the strain *Bacillus* sp. PUMB_17, a finding subsequently confirmed through cultivation in the Gerasimene medium. Under these cultural conditions, the supernatant of *Bacillus* sp. PUMB_17 was devoid of sphingomyelinase (SMase) and phosphatidylinositol-specific phospholipase C (PI-PLC) activities. The presence of PC-PLC was validated using thin-layer chromatography [31].

**Figure 1:**
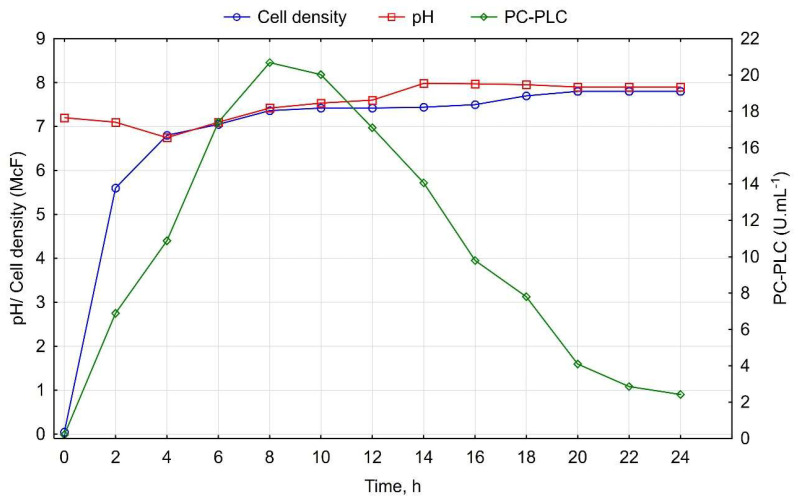
Dynamics of PC-PLC production during growth of *Bacillus paranthracis* PUMB_17.

The dynamics of enzymatic production were traced up to the 24th hour at 37 °C (Figure 1). Upon inoculation of the cultural medium with a subculture, the phospholipase C production commenced in the second hour and escalated rapidly, reaching a peak at the 8th hour (20.69 U/mL), after which it gradually declined to 2.42 U/mL at the 24th hour of cultivation. The culture’s growth was accompanied by changes in pH. Initially, the medium experienced slight acidification (pH 6.75 at the fourth hour), followed by subsequent alkalinization, reaching pH 7.9 at the 24th hour.

The highest PC-PLC secretion was achieved when the strain was cultivated at 37 °C at 100 rpm for 8 h in Gerasimene medium. The optimal amount of starter culture was 7.5% (*v*/*v*) of the inoculum. The PC-PLC activity of the supernatant under these conditions was 31 U/mL. The maximum PC-PLC secretion at the end of the exponential growth phase corroborates the literature findings on phospholipase enzyme production during the late logarithmic and early stationary phases in Gram-positive bacteria [32]. Similar results regarding the secretion of phospholipase C were noted in *C. perfringens* and *B. cereus* [26,33,34,35]. In Gram-negative bacteria, maximal enzymatic secretion occurs at a later stage in culture development, influenced by their physiological characteristics [36,37,38,39].

Most enzyme-secretion systems in bacteria can be regulated by catabolic or end products [38]. This implies that the synthesis of PLC may be delayed due to repression by certain components in the cultural environment. A reduction in substrates towards the end of the logarithmic and the beginning of the stationary phase may stimulate PLC expression [40]. Such fermentation, yielding phospholipase C, can be characterized as being growth-associated. Blanco and collaborators [41] described a similar enzyme synthesis profile in *B. subtilis*, acting as primary metabolites essential for bacterial culture development. Moreover, the dynamics of phospholipase enzyme production are not associated with cell autolysis in the culture. Bacterial phospholipids in intact cells were not hydrolyzed by extracellular enzymes [42]. According to some authors, phospholipase activity is strongly correlated with cell density. As the culture enters the exponential growth phase, enzymatic activity increases linearly, suggesting that phospholipase C expression may also be under the control of quorum-sensing systems [42,43]. Bacterial cell communication (quorum sensing) has been established to directly regulate gene expression in the bacterial community. In Gram-positive bacteria, cell communication relies on cytoplasmic sensors regulated by secreted and re-imported signaling peptides. Cell communication controls several essential functions in the *Bacillus cereus* group. For instance, it has been found that quorum sensing directly regulates the expression of phospholipase enzymes, and the virulence of *B. cereus* and *B. thuringiensis* species depends precisely on the expression of these enzymes [44].

### 3.2. In Silico Genomic Landscape Identifies PUMB_17 as a Bacillus paranthracis Strain

#### 3.2.1. Genome Overview and Species Identification

To explore the genomic context of the bacterial strain we used Nanopore ONT sequencing. Nanopore sequencing has several notable advantages when compared to traditional next-generation sequencing (NGS) technologies. One primary benefit is its capacity to produce longer reads, allowing researchers to acquire more comprehensive and contiguous genomic information. In contrast, short-read NGS technologies often struggle to resolve complex genomic regions and repetitive sequences. Nanopore sequencing, particularly with Oxford Nanopore Technologies (ONT), simplifies the assembly of bacterial genomes by providing such longer reads. It is important to highlight that ONT sequencing technology has undergone significant advancements in recent years (especially with R10 flow cells), leading to longer read lengths, lower error rates, and increased sequencing accuracy. Furthermore, the development of software-based calling algorithms has played a significant role in refining ONT sequencing data. Collectively, these factors are gradually enabling the independent use of ONT technology without the need for additional Illumina data.

With the achievements of whole-genome sequencing (WGS), there has been a significant increase in the number of publicly available genomes of *B. cereus s.l.* species submitted to the National Center for Biotechnology Information (NCBI) database [45,46,47]. High-resolution genomic data have enabled exploring previously uncharted areas in the *B. cereus s.l.* phylogeny, the data have also exacerbated the existing challenges arising from the absence of standardized taxonomic methods for identifying new *B. cereus s.l.* species.

In 2017, a study was published describing nine new *B. cereus s.l*. species, such as *B. albus*, *B. luti*, *B. mobilis*, *B. nitratireducens*, *B. pacificus*, *B. paramycoides*, *B. paranthracis*, *B. proteolyticus*, and *B. tropicus*, effectively doubling the number of published species within this group [10]. It is crucial to note that this study used a species threshold of 95–96% ANI for strain identification, which was recently adopted for improving the taxonomic assignment in prokaryotic genomes [17].

**Figure 2:**
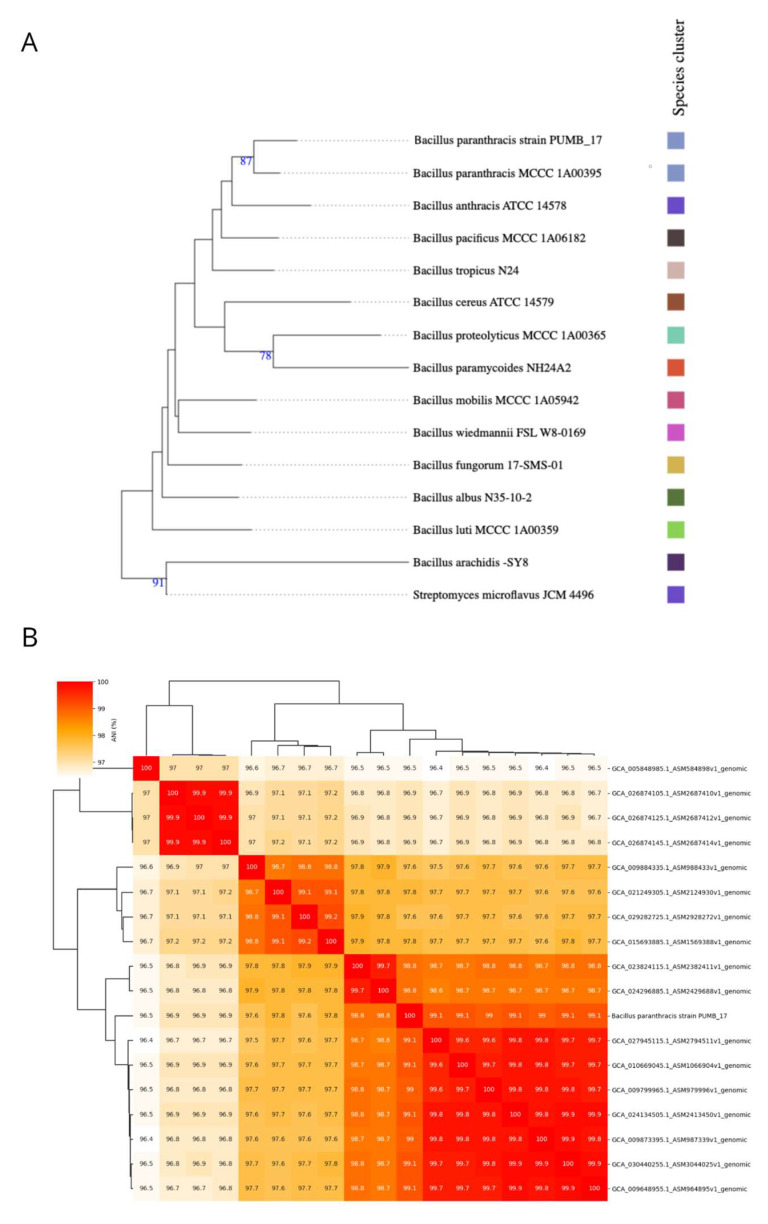
Species identification of the PUMB_17 genome: (**A**) whole-genome phylogenetic tree generated using TYGS database and (**B**) ANI heatmap calculated between the PUMB_17 genome and the 17 complete *B. paranthracis* genomes available in NCBI.

In the case of the PUMB_17 strain, the genome similarity was evaluated using ANI, which was calculated between the PUMB_17 strain genome and the 17 complete *B. paranthracis* genomes available in NCBI (Figure 2B). The PUMB_17 strain showed an ANI value ranging from 96.5% to 99.1%. Further, phylogenomic analysis using genome–genome comparisons in TYGS revealed that the PUMB_17 strain is clustered with other representative strains in the database (Figure 2A). Furthermore, upon uploading the genome in NCBI, its pipelines also identified the species as *B. paranthracis*. MOB-Typer results showed that the PUMB_17 strain possessed two mobilizable plasmids having MOBC and MOBV relaxase types.

**Figure 3:**
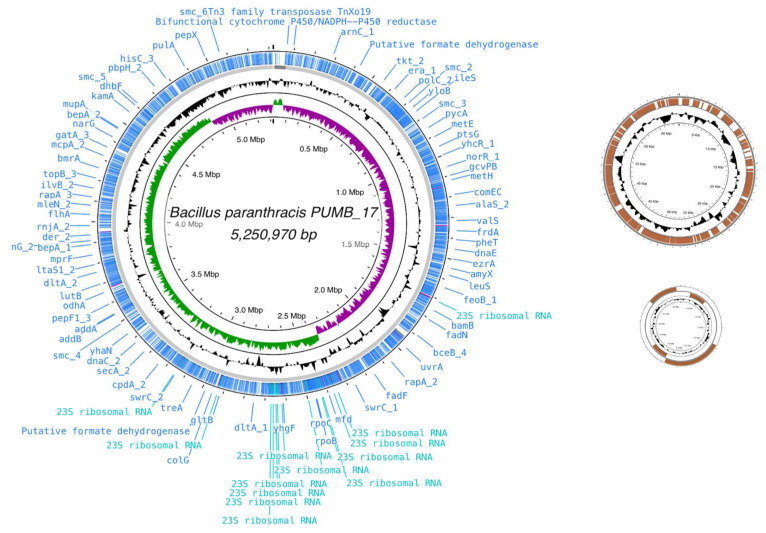
Genomic map of the *B. paranthracis* PUMB_17 genome visualized using Proksee tool—bacterial chromosome and two plasmids.

There are 17 complete genomes of *B. paranthracis* publicly available in NCBI, the size of which ranges from 5.1 M to 6.0 M. In this regard, the complete genome of *B. paranthracis* strain PUMB_17 fits within that range. It contains a single circular chromosome of 5,250,970 bp (coverage ×80) with a guanine–cytosine (GC) ratio of 35.49, with two plasmids with size 64,250 bp and 5,845 bp, respectively (Figure 3). The complete genomic sequences of *B. paranthracis* PUMB_17 have been submitted to the NCBI submission portal under accession numbers CP129604 (chromosome), CP129603, and CP129602 (plasmids).

#### 3.2.2. Genome Annotation

**Figure 4:**
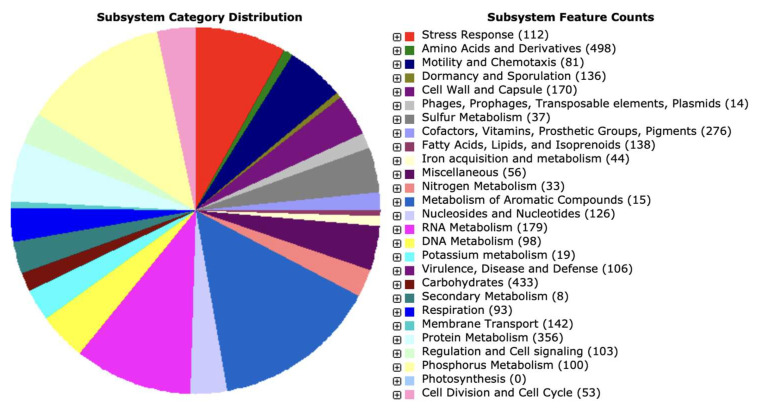
Distribution of *B. paranthracis* PUMB_17 subsystem gene functions. The pie chart shows the count of each subsystem feature and subsystem coverage.

We utilized the RAST server for genome annotation and supplemented the analysis with the findings from BlastKOALA. In total, we identified 5328 genes, consisting of 5186 protein-coding sequences (CDSs) and 142 RNA genes (comprising 39 rRNAs, 103 tRNAs, and 5 ncRNAs). Among the predicted CDSs, 3978 genes (approximately 77%) were associated with annotated functions, while 1207 genes (about 23%) remained hypothetical or had unknown functions. RAST analysis revealed that these proteins participate in 469 subsystems, as illustrated in Figure 4. A comprehensive table containing both RAST and BlastKOALA annotations is provided in Supplementary Table S1.

The genome annotation revealed that the majority of genes were assigned to the subsystem category of amino acids and their derivatives (498 genes); followed by carbohydrates (433 genes); protein metabolism (356 genes); cofactors, vitamins, prosthetic groups, and pigments (276 genes); nucleosides and nucleotides (126 genes); dormancy and sporulation (136 genes); cell wall and capsule (170 genes); RNA metabolism (179 genes); DNA metabolism (98 genes); fatty acids, lipids, and isoprenoids (138 genes); stress response (112 genes); motility and chemotaxis (81 genes); membrane transport (142 genes); respiration (93 genes); virulence, disease, and defense (106 genes), phosphorus metabolism (100); and others (Figure 4).

CAZymes, responsible for catalyzing the biosynthesis, modification, and breakdown of carbohydrates and glycoconjugates, fall into five distinct categories, (a) glycoside hydrolases (GHs), (b) glycosyl transferases (GTs), (c) polysaccharide lyases (PLs), (d) carbohydrate esterases (CEs), and (e) auxiliary activities (AAs). Auxiliary activities encompass enzymes that facilitate hydrolysis, formation, non-hydrolytic cleavage of glycosidic bonds, hydrolysis of carbohydrate esters, and enzymes that cleave complex carbohydrates. Additionally, CAZymes include carbohydrate-binding modules (CBMs), which refer to specific amino acid sequences within a CAZyme exhibiting carbohydrate-binding activity. Such enzymes can be of great importance regarding production and industrial use, especially when they are extracellular. We used dbCAN3 to scan the proteome of PUMB_17 for CAZymes. We found 44 glycoside hydrolases, 51 glycosyl transferases, 6 proteins with auxiliary activities, and 21 with carbohydrate-binding modules. Twenty-one CAZymes pose as signal peptides, which had various substrates such as chitin (QRA03_02980, QRA03_14680, QRA03_25650, QRA03_25800), alpha-galactan (QRA03_11915), cellulose/xylan (QRA03_14680), glycan (QRA03_22285), beta-glucan (QRA03_25360), starch (QRA03_25360), and xyloglucan (QRA03_27475), among others. Two proteins contained the CBM50 module (QRA03_03130, QRA03_20010), which shows antifungal activity against various pathogenic fungi and is found in association with enzymes and proteins related to cell wall degradation [48,49].

To further explore the secretome of PUMB_17 (not only for carbohydrate-active enzymes), we also used the PSORTb 3 tool to predict the extracellular proteins. Of all protein-coding sequences, 85 entries were shown to be extracellular and 26 were with unknown functions and had no annotations. Some of the extracellular proteins include PI-PLC, PC-PLC, SMase C, manganese superoxide dismutase, bacillolysin, microbial collagenase, metalloprotease, peptidase, the M23/M37 family, alkaline serine protease, the subtilase family, triacylglycerol lipase, and alkaline phosphatase, among others.

*B. cereus s.l.*, like many other multi-drug resistant microorganisms, exhibits varying antibiotic resistance mechanisms across different strains. Typically, they demonstrate resistance to penicillin and cephalosporins, primarily attributed to the production of beta-lactamase [50,51]. Results from CARD and Multires databases screening using the Abricate tool gave output to four antimicrobial resistance genes encoded in the chromosome, as follows: *bcII* (QRA03_01595) and *bla1* (QRA03_24455) genes related to Class B and Class A beta-lactamases resistance; *fosB* gene (QRA03_22435); and finally, the genome of PUMB_17 harbored the *satA* gene, related to streptothricin. It was noted that all the identified antimicrobial resistance (AMR) genes were encoded in the chromosome. Notably, we did not find any genes linked to resistance against other antibiotics, such as tetracycline, vancomycin, or streptogramin, which have been reported for other members of the *B. cereus* s.l. group [52,53,54]. Genes related to beta-lactams and fosfomycin resistance are also observed in all 17 strains available with the full genome at NCBI, indicating that these genes are members of the core genes for the *B. paranthracis*.

The identified *B. paranthracis* strain PUMB_17 was observed to harbor some potential virulence genes. Among these genes, *nheA*, *nheB*, and *nheC* are responsible for producing a non-hemolytic enterotoxin consisting of *NheA*, a cytolytic protein, and two associated proteins, *NheB* and *NheC*. These associated proteins usually serve to enhance the cytolytic protein’s biological effectiveness, as is also observed in other species strains. In contrast, PUMB_17 lacked the virulence genes *hblA* and *cytK2*, observed in recently identified pathogen strains *B. paranthracis* 4M, 1702, and 1710 [55]. In the protease category, immune inhibitor A metalloprotease (*inhA*, QRA03_15915) was detected; this gene assists in surviving the host environment conditions. The VFDB also shows the presence of a non-ribosomal peptide synthetase (NRPS) gene cluster, putatively resulting in the production of a bacillibactin-like siderophore (*dhbA/B/C/D/E/F*, QRA03_23840-QRA03_23860), which is the most common siderophore in Gram-positive bacteria, produced by *Bacillus subtilis*, *B. cereus*, *B. anthracis*, *B. thuringiensis*, and *B. amyloliquefaciens*. [56,57,58]. Since the genome does not possess genes coding for cereulide synthetase, enterotoxin FM, and cytotoxin K, which are commonly present in food poisoning pathogens such *B. cereus* and *Bacillus thuringiensis* [59], PUMB_17 may lack the capacity to be a pathogen. Several studies with similar findings reported that some strains of *B. paranthracis* (ICIS-279 and MHSD3) may even hold a potential for probiotics [11,59].

Phospholipases and sphingomyelinases are also considered virulence factors of many bacteria, including those belonging to the *B. cereus* group. The genome of PUMB_17 harbors the genes for PLCs—*plcA* gene (QRA03_03125) and *cerA* with *cerB/sph* genes in a cluster (QRA03_15935-QRA03_15940).

We have conducted a pan-genome analysis of all available strains of *B. paranthracis* in the NCBI database. The core genes of the strains represented 33.02% (3057 with pan-genome size: 9208 clusters), of which *cerA* encodes for PC_PLC and cerB/sph—for SMAse C (QRA03_15935, QRA03_15940). The *plcA* encoding PI_PLC (QRA03_03125) was present in 16 of 17 strains. BLAST analysis of the PC-PLC protein sequence (283 aa) of *cerA* gene shows that it is identical with the multispecies record WP_000731014.1, which includes various species of the *Bacillus cereus* group (mainly *B. paranthracis* and also *B. tropicus, B. pacificus*, and unclassified *B. cereus* group members).

Originally isolated from sediment in the Pacific Ocean [10], *Bacillus paranthracis* has been identified in human feces, with documented associations to instances of diarrhea [60,61]. Additionally, the bacterium has been implicated in cases of bovine mastitis, underscoring its potential pathogenicity [55]. However, the characterization of *B. paranthracis* remains incomplete, with a paucity of publications employing whole-genome sequencing to explore its strains comprehensively.

Several extensively analyzed strains have been derived from diverse sources, such as fermented foods [62], meat and poultry products [63], and plants [64]. Investigations utilizing whole-genome sequencing techniques have unveiled strains of *B. paranthracis* exhibiting potential probiotic characteristics [64] and demonstrating biodegradation capabilities [65]. Nonetheless, the current body of literature on *B. paranthracis* strains using whole-genome sequencing remains limited, emphasizing the need for further research to elucidate this bacterial species’ full spectrum of characteristics and potential applications.

### 3.3. Purification of PC-PLC from PUMB_17

**Figure 5:**
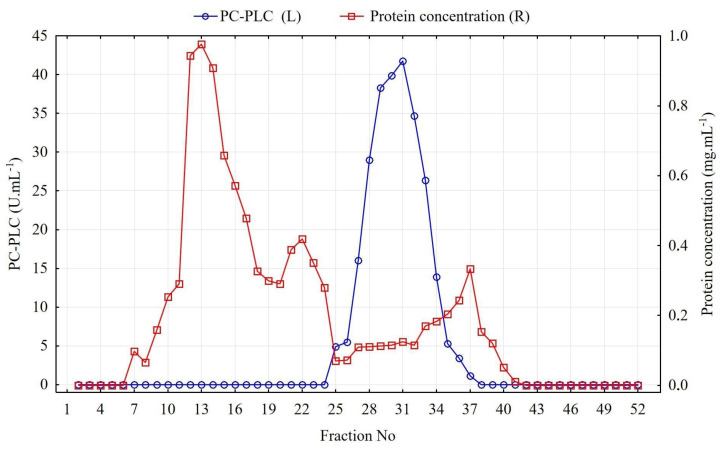
Size exclusion chromatography of a concentrated supernatant (ultrafiltrate) from *B. paranthracis* PUMB_17 culture.

PC-PLC was isolated and purified from a cell-free supernatant obtained from the *B. paranthracis* PUMB_17 strain. The total protein content in the supernatant was 1008.65 mg, with 3537 U total PC-PLC activity (Table 1). After ultrafiltration through a nitrocellulose membrane with a cutoff of 10 kDa, the protein in the supernatant was concentrated 6-fold, and the enzyme activity yield was almost fully recovered—87.4% (3091 U). These results showed that the supernatant was concentrated and some degree of purification of the cell-free extract was achieved. This concentrate was additionally purified using size exclusion chromatography. A major part of the total protein content eluted from the column with the first major peak showed no PC-PLC activity (Figure 5). The enzyme was eluted in a single peak of activity in 13 fractions from 219 mL to 310 mL. The PC-PLC was purified 50.73-fold with a 60.65-fold reduction in total protein concentration. The PC-PLC yield was 50.73% (Table 1).

**Figure 6:**
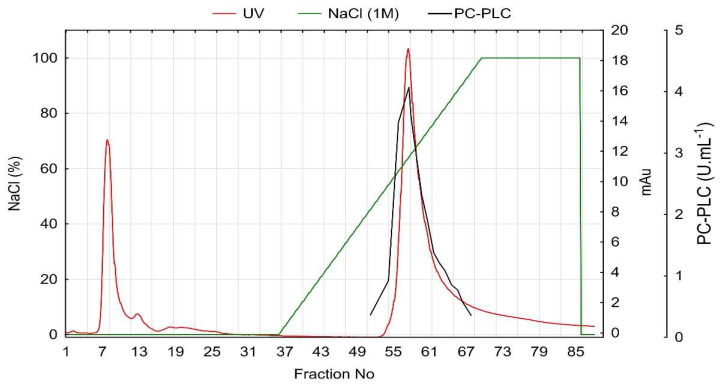
Anion exchange chromatography of PUMB_17 PC-PLC.

All fractions with established PC-PLC activity were pooled in a single sample, which was additionally purified using anion exchange chromatography (AEX). Two main protein peaks were observed, but only the second peak had PC-PLC activity. The enzyme was eluted at 325 mM sodium chloride and was recovered in 13 fractions with a total volume of 39 mL. The total protein concentration was reduced 155-fold, the PC-PLC was purified 54.45-fold, and the achieved yield was 35.18% (Figure 6 and Table 1).

Phospholipase C has been purified to a homogeneous state from various Gram-positive and Gram-negative bacteria [66]. Predominant purification procedures typically involve initial protein concentration in the supernatant, achieved through precipitation with ammonium sulfate or saturation with organic solvents (ethanol or acetone) or acids (usually saline), followed by various chromatographic methods, such as gel filtration, ion-exchange, and affinity chromatography [42,67].

For purification of PC-PLC from a strain of *B. thuringiensis* isolated from Egyptian soil, Aboulwafa et al. [68] reported a salting-out step with ammonium sulfate and molecular-sieve chromatography on Sephadex G-75. Eddelech and coworkers [69] have purified PC-PLC from a Tunisian strain of *B. thuringiensis* using ammonium sulfate precipitation, followed by Sephadex G-75 and Q-Sepharose column chromatography.

Our purification scheme differs from those described in the literature in the choice of method for concentrating the supernatant. The applied ultrafiltration step allows for a 5.75-fold purification and a yield exceeding 85%.

Samples of the supernatant, ultrafiltrate, and purified material after size exclusion chromatography, as well as a sample of the purified PC-PLC after AEX were analyzed using SDS–PAGE electrophoresis to determine the protein profiles and purity of the various preparations. The SDS–PAGE analysis of the supernatant sample and ultrafiltrate (Figure S1) showed that *B. paranthracis* secretes a rich variety of proteins with molecular weights ranging from 10 kDa to more than 100 kDa. This observation confirms the results of Han et al. [70] who established that species of the *Bacillus* secrete more than 40 different extracellular enzymes.

**Figure 7:**
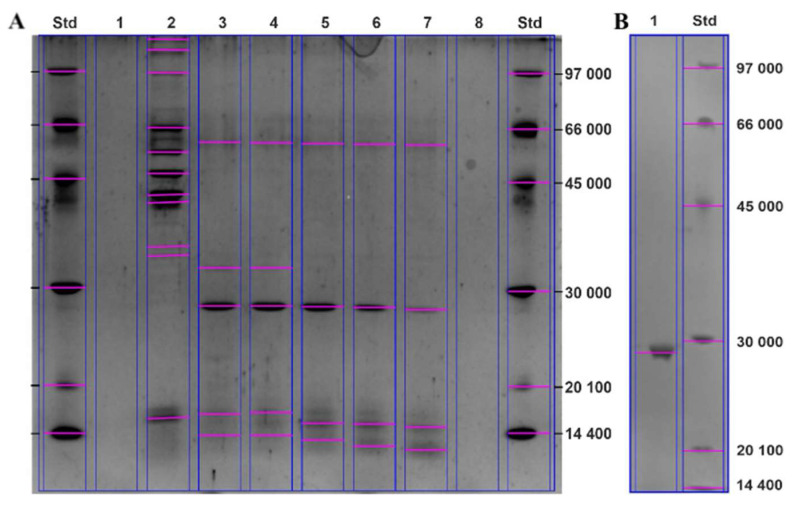
SDS–PAGE analysis of samples of purified PC-PLC produced by *B. paranthracis* PUMB_17. SDS–PAGE gel after size exclusion chromatography (**A**), SDS–PAGE gel after AEX (**B**). Fractions with highest PC-PLC activity ((**A**): 3, 4, 5, 6), fraction with high protein content, but without any PC-PLC activity ((**A**): 2).

The conducted size exclusion chromatography led to a significant reduction in the total protein concentration of 16.63 mg/mL. A major part of the remaining protein in the fractions with high PC-PLC activity had a molecular weight of 25–30 kDa (Figure 7A). However, at this stage at least two high molecular proteins with molecular weight of about 66 kDa were also present, as well as some low molecular fractions (14 kDa). The ion exchange chromatography allowed further purification of the PC-PLC to a single protein showing well-exhibited enzyme activity with a molecular weight of 25–30 kDa (Figure 7B).

#### PC-PLC Zymography

**Figure 8:**
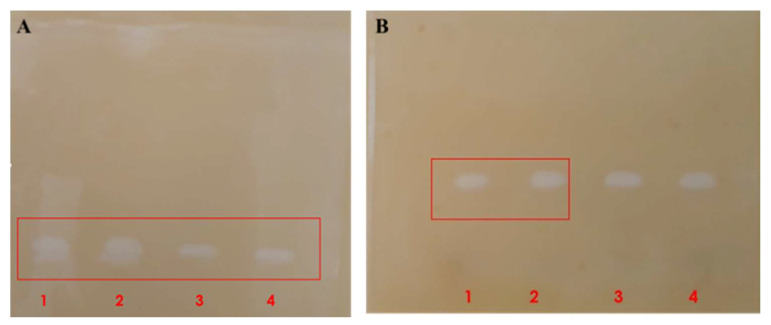
Zymography of samples containing PC-PLC produced by *B. paranthracis* PUMB_17. Supernatant ((**A**): 1, 2), ultrafiltrate ((**A**): 3, 4) and samples after performing size exclusion chromatography ((**B**): 1, 2, 3).

Zymograms were used to detect the PLC activity of SDS–PAGE bands with apparent molecular mass, visualized on the silver-stained SDS–PAGE. A single diglyceride band on the egg yolk agar plate, corresponding to the band with lecithinase activity, was detected in the ultrafiltrate fraction (Figure 8A) and in the partially purified enzyme fraction collected after the size exclusion chromatography (Figure 8B). The diglyceride band corresponds to the proteins with molecular mass 25–30 kDa, on the SDS–PAGE (Figure 7).

The obtained results align with the genomic data, specifically regarding the PC-PLC gene (*cerA*). This gene encodes a protein of 32.39 kDa (283 amino acids), identical to the multispecies *Bacillus* accession number WP_000731014.1. As previously reported by other studies, the secreted monomeric protein exhibits a lower molecular mass due to the maturation process, involving two distinct steps. Firstly, the 24-amino acid pre-sequence undergoes cleavage by type I signal peptidases. Subsequently, an additional processing step occurs, wherein unidentified extracellular proteases remove the 14-amino acid pro-sequence [71]. This intricate process results in the formation of the functional enzyme with a total of 245 amino acids corresponding to the 25–30 kDa, on the SDS–PAGE.

## 4. Conclusions

The species belonging to the genus *Bacillus* are widely distributed due to their exceptional adaptability, flexible metabolism, and capacity to utilize various substrates. The produced phospholipase C functions naturally in the accumulation and mobilization of energy reserves within the cell. The phospholipases and the products resulting from the hydrolysis of phospholipids find utility in the production of detergents, as well as in the food and pharmaceutical industries, among others [72,73,74].

In addition to the aforementioned insights into the versatility of the *Bacillus* species and the industrial applications of phospholipases, our study reports *Bacillus paranthracis* as a competent producer of PC-PLC, for the first time. The PUMB_17 strain exhibits extracellular PC-PLC production with high specific activity during the late exponential growth phase. We can speculate that PC-PLC is resistant to proteolytic degradation, which may facilitate the preparative-scale enzyme production process. Employing a simple three-step purification scheme involving ammonium sulfate precipitation, gel filtration, and anion exchange chromatography, a highly purified preparation of PC-PLC from *B. paranthracis* PUMB_17 was obtained and confirmed by SDS electrophoresis and zymography. To provide a comprehensive understanding of the genomic characteristics of PUMB_17, we employed nanopore-based whole-genome sequencing, followed by meticulous genome annotation. This systematic genomic exploration serves as a pivotal step in unravelling the previously understudied and recently documented *Bacillus paranthracis*.

## Figures and Tables

**Table 1:** Anion exchange chromatography purification of PC-PLC produced by PUMB_17.

	Activity (U)	Protein (mg)	Specific Activity (U/mg)	Purification (Fold)	Yield (%)
Supernatant	3537.07	1008.64	3.51	-	100.00
Ultrafiltrate	3091.40	171.75	18.00	5.13	87.40
Sephadex G75	1794.45	16.63	107.89	30.77	50.73
HiPrep DEAE	1244.23	6.52	190.93	54.45	35.18

## Data Availability

Data is contained within the article and Supplementary Materials.

## Supplementary Materials

The following supporting information can be downloaded at: https://www.mdpi.com/article/10.3390/cimb46030158/s1, Figure S1: SDS-PAGE analysis of samples of supernatant and ultrafiltrate; Table S1: Genome annotation.
