# Supplementary figures and images for: Exploring the Genomic Landscape of Bacillus paranthracis PUMB_17 as a Proficient Phosphatidylcholine-Specific Phospholipase C Producer
Source: Curr Issues Mol Biol. 2024 Mar 14;46(3):2497–513. doi: 10.3390/cimb46030158 (PMC10969478; doi:10.3390/cimb46030158)

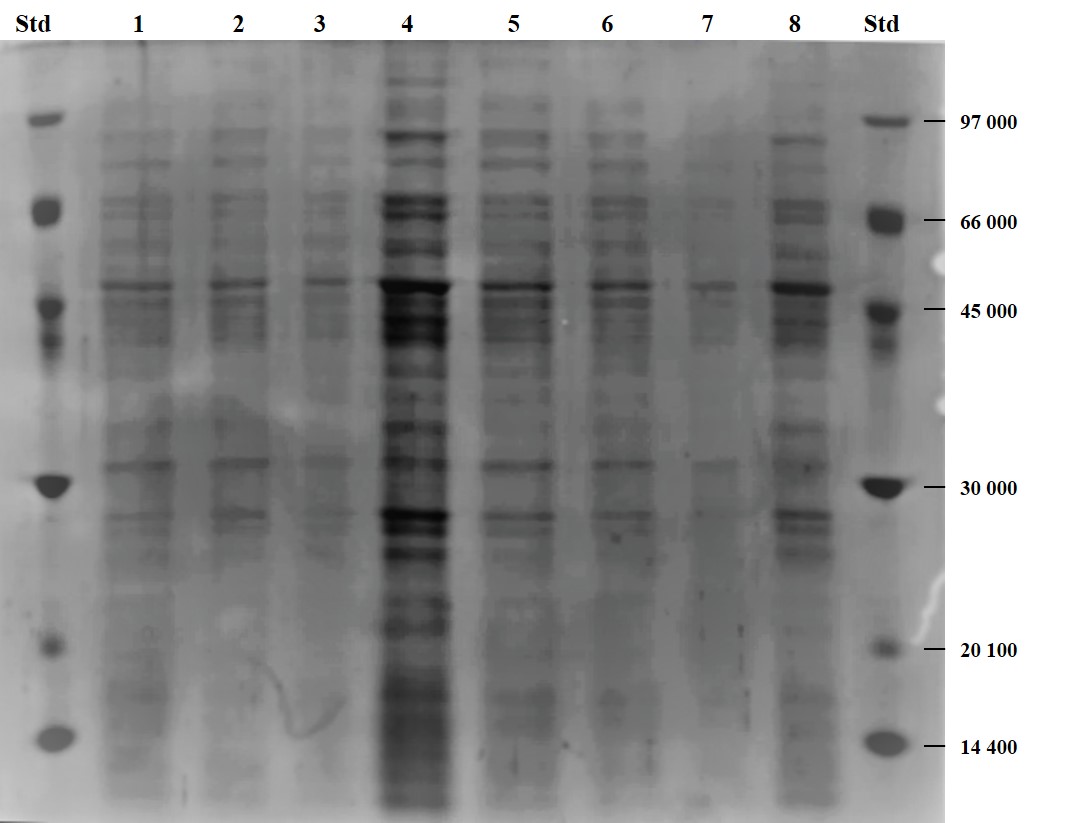

Supplement: Supplementary file 1 [file cimb-46-00158-s001.zip › Figure S1.jpg]
